# Evaluation of clinical datasets in fully automatic planning assist system for cardiac magnetic resonance imaging

**DOI:** 10.1186/1532-429X-15-S1-P33

**Published:** 2013-01-30

**Authors:** Shuhei Nitta, Taichiro Shiodera, Tomoyuki Takeguchi, Shigehide Kuhara, Kenichi Yokoyama, Rieko Ishimura, Toshiaki Nitatori

**Affiliations:** 1 Corporate Research & Development Center, Toshiba Corporation, Kawasaki, Japan; 2 MRI Systems Division, Toshiba Medical Systems Corporation, Otawara, Japan; 3 Department of Radiology, Kyorin University, Faculty of Medicine, Mitaka, Japan

## Background

Planning assist systems for cardiac MR examinations are necessary for easier operation and shorter examination times. Slice alignment systems [1] have played an important role in achieving these objectives. We propose a new automatic planning assist system for couch adjustment, local shimming, and axial multislice imaging as the input to a slice alignment system [2]. The new system employs an atlas-based segmentation technique using single scout volume data. In the present study, the accuracy and robustness of the proposed method were evaluated based on more than 50 datasets including clinical data, and the results were compared against the degree of interobserver error in manual annotation.

## Methods

An ECG-non-gated 3D fast field echo (FFE) single volume covering the entire chest area was acquired using a 1.5T MRI scanner (Excelart VantageTM powered by Atlas, Toshiba Medical Systems) during a single breath-hold with TR/TE = 3.7/1.3, FOV = 500 x 350 x 350 mm^3^ (coronal slab), and readout/phase/slice encode steps = 256/64/35 in an acquisition time of approximately 9 seconds. Our proposed method is based on a registration technique composed of several steps to achieve good accuracy with acceptable processing cost. First, the input volume is normalized in contrast by a histogram expansion. Then, the volume is transformed to match a prepared model volume with manual annotation of the heart region, permitting the heart region of the input data to be located. Finally, the result is modified by several image processing techniques. Accuracy was assessed by measuring the Euclidean distances of the six sides of the circumscribed cuboids of the cardiac area obtained by our method and by manual annotation, and evaluation was performed by comparison with the differences between two manual annotations as a measure of interobserver error.

## Results

The proposed method successfully segmented the entire heart region for 48 datasets from 15 healthy volunteers and 51 datasets from 32 patients. The processing time was approximately 1.6 seconds (2.5 GHz CPU, single-thread processing). The average distance error for the left, right, anterior, posterior, head, and foot sides were 3.91±3.28, 3.10±2.43, 4.31±3.32, 6.80±5.76, 10.42±8.96, and 6.15±5.12 mm, respectively. The interobserver errors for 15 datasets from 15 healthy volunteers and 32 datasets from 32 patients were 3.61±2.68, 3.25±3.09, 4.31±4.39, 5.77±3.97, 13.22±8.52, and 4.42±3.18 mm, respectively.

## Conclusions

We propose a new cardiac alignment system. The results showed that, even in patient data, the entire heart region could be detected by our method almost as accurately as by manual annotation. The proposed method combined with our previous work [2] should prove to be clinically useful as a fully automatic planning assist system for cardiac MRI.

## Funding

No funding was received for this research.

**Figure 1:**
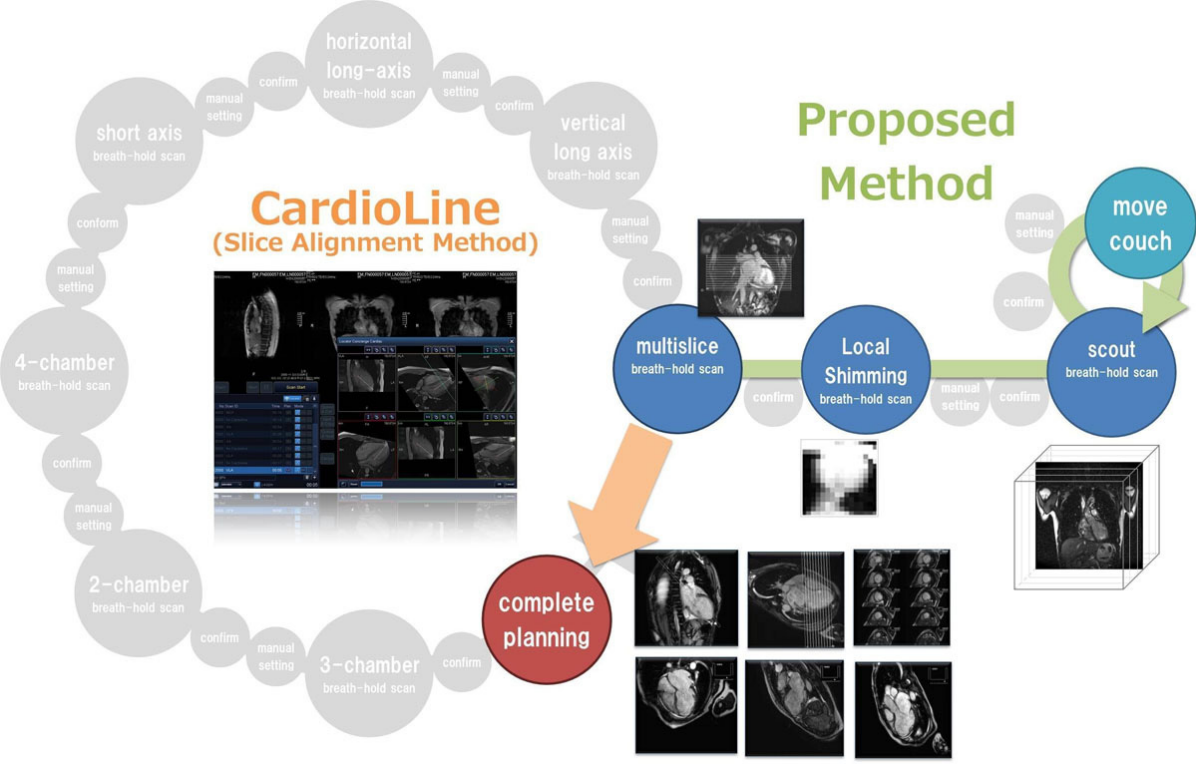
Automatic planning assist system for cardiac MRI exams.

**Figure 2:**
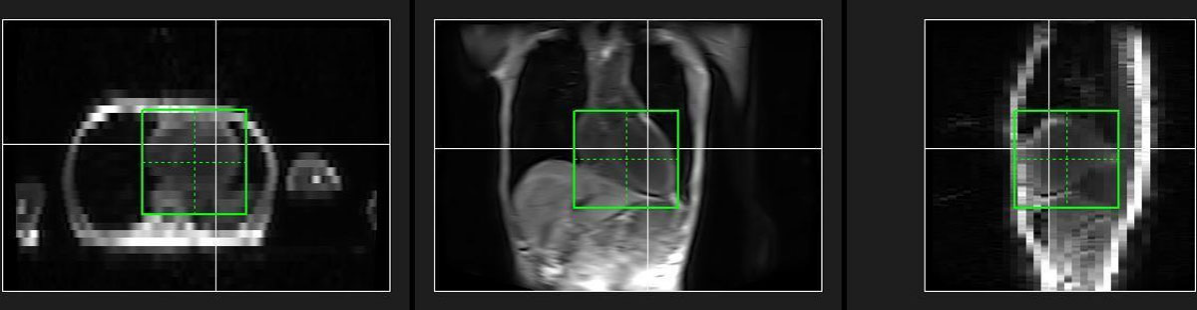
Example of detection results for actual clinical data.
